# Epileptiform activity influences theta-burst induced LTP in the adult hippocampus: a role for synaptic lipid raft disruption in early metaplasticity?

**DOI:** 10.3389/fncel.2023.1117697

**Published:** 2023-05-09

**Authors:** José D. Carvalho-Rosa, Nádia C. Rodrigues, Armando Silva-Cruz, Sandra H. Vaz, Diana Cunha-Reis

**Affiliations:** ^1^Departamento de Química e Bioquímica, Faculdade de Ciências, Universidade de Lisboa, Lisbon, Portugal; ^2^BioISI–Biosystems and Integrative Sciences Institute, Faculdade de Ciências, Universidade de Lisboa, Lisbon, Portugal; ^3^Instituto de Medicina Molecular João Lobo Antunes, Faculdade de Medicina, Universidade de Lisboa, Lisbon, Portugal; ^4^Instituto de Farmacologia e Neurociências, Faculdade de Medicina, Universidade de Lisboa, Lisbon, Portugal

**Keywords:** seizures, epileptiform activity, bicuculline, low Mg^2+^, long term potentiation (LTP), mesial temporal lobe epilepsy (MTLE), lipid rafts

## Abstract

Non-epileptic seizures are identified as a common epileptogenic trigger. Early metaplasticity following seizures may contribute to epileptogenesis by abnormally altering synaptic strength and homeostatic plasticity. We now studied how *in vitro* epileptiform activity (EA) triggers early changes in CA1 long-term potentiation (LTP) induced by theta-burst stimulation (TBS) in rat hippocampal slices and the involvement of lipid rafts in these early metaplasticity events. Two forms of EA were induced: (1) interictal-like EA evoked by Mg^2+^ withdrawal and K^+^ elevation to 6 mM in the superfusion medium or (2) ictal-like EA induced by bicuculline (10 μM). Both EA patterns induced and LTP-like effect on CA1 synaptic transmission prior to LTP induction. LTP induced 30 min post EA was impaired, an effect more pronounced after ictal-like EA. LTP recovered to control levels 60 min post interictal-like EA but was still impaired 60 min after ictal-like EA. The synaptic molecular events underlying this altered LTP were investigated 30 min post EA in synaptosomes isolated from these slices. EA enhanced AMPA GluA1 Ser831 phosphorylation but decreased Ser845 phosphorylation and the GluA1/GluA2 ratio. Flotillin-1 and caveolin-1 were markedly decreased concomitantly with a marked increase in gephyrin levels and a less prominent increase in PSD-95. Altogether, EA differentially influences hippocampal CA1 LTP thorough regulation of GluA1/GluA2 levels and AMPA GluA1 phosphorylation suggesting that altered LTP post-seizures is a relevant target for antiepileptogenic therapies. In addition, this metaplasticity is also associated with marked alterations in classic and synaptic lipid raft markers, suggesting these may also constitute promising targets in epileptogenesis prevention.

## Graphical Abstract

Altered long term potentiation (LTP) following *in vitro* epileptiform activity and major synaptic molecular alterations triggered. 
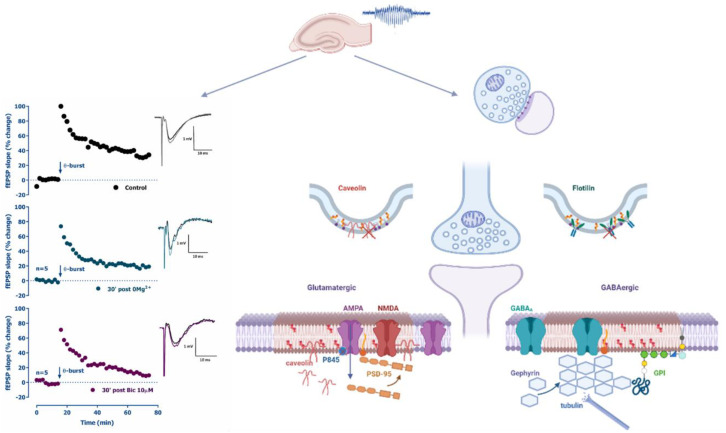

## Introduction

Epilepsy, a multifaceted disease characterized by the development of recurrent, unprovoked seizures, is a neurological disorder that affects over 65 million people worldwide. Its high-incidence and high-prevalence (6.4 cases per 1,000 persons) is nevertheless minored by its high mortality owing to accidents, *status epilepticus* or sudden unexpected death in epilepsy, often occurring during seizures (Devinsky et al., 2018). Being the most frequent, chronic, severe neurological disease it is a huge burden for public health systems (Moshé et al., 2015; Devinsky et al., 2018; Vezzani et al., 2019; Cunha-Reis et al., 2021) and since over one third of epilepsy cases are refractory to treatment with multiple antiseizure drugs (ASDs), it is imperative to identify new therapeutic targets.

Epileptogenesis, the process by which healthy brain networks develop enhanced susceptibility to generate spontaneous recurrent seizures leading to the development of epilepsy (Pitkänen et al., 2015; Vezzani et al., 2019), is currently perceived as a continuous and progressive process extending far beyond the latent period. Yet, the early pathophysiological mechanisms involved in epileptogenesis are still for the most part unknown and tackling epileptogenesis is an unmet clinical need (Pitkänen et al., 2015; Cunha-Reis et al., 2021). One key element is that different triggers likely lead to different seizure-onset, which will ultimately determine its first line of treatment (Sloviter, 2017). As such, establishing therapeutic targets in this early phase of epileptogenesis requires a better knowledge of these mechanisms.

Mesial temporal lobe epilepsy (MTLE) involves seizures typically arising in the hippocampus or other mesial temporal lobe structures. Only about 11–26% of patients with MTLE with hippocampal sclerosis achieve complete seizure control under pharmacological treatment with existing ASDs. As such, patients often require neurosurgical resection of the epileptic focus as a last resource to ameliorate seizure recurrence and to prevent fast worsening of the disease (Liu et al., 2012; Kuang et al., 2014; Thom, 2014). The etiology of MTLE epileptogenesis in still unknown, yet putative precipitating events such as trauma, complex febrile seizures, status epilepticus, inflammatory insults, or ischemia have been implicated. Such events may trigger epileptogenesis by generating aberrant synaptic plasticity/neuronal excitability, excitotoxicity, secondary non-convulsive *status epilepticus*, neuroinflammation and generation of reactive oxygen species that develop during a silent period, when spontaneous seizures do not occur (Thom, 2014; Gambardella et al., 2016; Sloviter, 2017), but their exact chronology it is not often easy to establish partly due to the multitude of putative epileptogenic events.

Within the first days of disease progression, enhanced neurogenesis and axonal sprouting accompany changes in neuronal excitability and synaptic plasticity driving the formation of novel synaptic connections and aberrant neural networks (Beck and Yaari, 2008). Synaptic plasticity, the ability of synapses to undergo long-lasting, activity-dependent bidirectional changes in the strength of synaptic communication, can be mediated by altering the gain of stimulus-release coupling at the presynaptic component or by changes in the type, number, or properties of the neurotransmitter receptors and their coupling to the intracellular signaling machinery at the postsynaptic level (Bliss and Collingridge, 2019; Cunha-Reis and Caulino-Rocha, 2020). It has been hypothesized that such changes may occur maladaptively immediately following non-epileptic seizures, thus triggering epileptogenesis (Avanzini et al., 2014), but this has not so far been thoroughly investigated.

Lipid rafts are membrane microdomains involved in synaptic receptor clustering, synaptic signaling, synaptic vesicle recycling and neurotransmitter release (Allen et al., 2007) and may have a crucial role in both synaptic and homeostatic plasticity under such pathological conditions (Sebastião et al., 2013). Synaptic lipid raft markers include both classic raft-associated proteins like caveolin-1 and flotillin-1, characteristic of caveolae and planar lipid rafts, respectively, and structural postsynaptic receptor anchoring proteins like PSD-95, present at glutamatergic synapses, and gephyrin, distinctive of GABAergic synapses (Tulodziecka et al., 2016; Papadopoulos et al., 2017; Bai et al., 2021). The latter two are also recognized as important NMDA, AMPA, and GABA_*A*_ membrane-anchoring proteins, respectively.

*In vitro* models of ictogenesis have provided important insights into the therapeutic potential of several candidate anti-seizure drugs (Gloveli et al., 1995; Debanne et al., 2006; Antonio et al., 2016; Dulla et al., 2018). In this paper we used such *in vitro* models of epileptiform activity to evaluate the time-course of changes in LTP occurring within 30 min to 1 h 30 m following seizures. LTP was evoked *in vitro* by theta burst stimulation (TBS), a sequence of electrical stimuli that mimic CA1 pyramidal cell burst firing as occurring during the hippocampal theta rhythm (4–10 Hz), an EEG pattern linked to hippocampal memory storage in rodents and involves the recruitment of GABAergic mechanisms (Vertes, 2005; Larson and Munkácsy, 2015; Artinian and Lacaille, 2018; Rodrigues et al., 2021). We characterized also the synaptic molecular alterations in AMPA receptors potentially underlying impaired LTP. In addition, its association with modified lipid raft dynamics, an important requirement for several molecular mechanisms involved in synaptic plasticity, was also investigated. A preliminary account of some of the results has been published as an abstract (Carvalho-Rosa and Cunha-Reis, 2019).

## Materials and methods

All animal procedures and protocols were performed according to ARRIVE guidelines for experimental design, analysis, and their reporting. Animal housing and handing was performed in accordance with the Portuguese law (DL 113/2013) and European Community guidelines (86/609/EEC and 63/2010/CE). The experiments were performed on hippocampal slices (400 μm thick), cut perpendicularly to the long axis of the hippocampus, obtained from young-adult (6–7 weeks old) male outbred Wistar rats (Harlan Iberica, Barcelona, Spain) as previously described (Aidil-Carvalho et al., 2017). Female rats were not used due to hormonal influences on LTP (Warren et al., 1995; Good et al., 1999). Rats were anesthetized with halothane, decapitated, and both hippocampi were extracted in ice-cold artificial cerebrospinal fluid (aCSF), composition in mM: NaCl 124, KCl 3, NaH_2_PO_4_ 1.25, NaHCO_3_ 26, MgSO_4_ 1, CaCl_2_ 2, glucose 10, and oxygenated with a 95% O_2_ - 5% CO_2_ mixture.

This is known as paired-pulse facilitation (PPF) and is believed to result from the presence of residual calcium generated by the first stimulation in glutamatergic terminals, that results in an enhanced release of neurotransmitter in response to the second stimulation (Zucker, 2003) at CA3–CA1 synapses, where the release probability is low under the current stimulation conditions (Wu and Saggau, 1994; Debanne et al., 1996). PPF in the CA1 area of the hippocampus has also been attributed to paired-pulse depression in the fast inhibitory postsynaptic potential (IPSP) (Nathan and Lambert, 1991) believed to result from GABAB autoreceptor-mediated depression of neurotransmitter release at interneuron–pyramidal cell synapses (Davies et al., 1990; Lambert and Wilson, 1994).

### Electrophysiological recordings and induction of epileptiform activity

**FIGURE 1:**
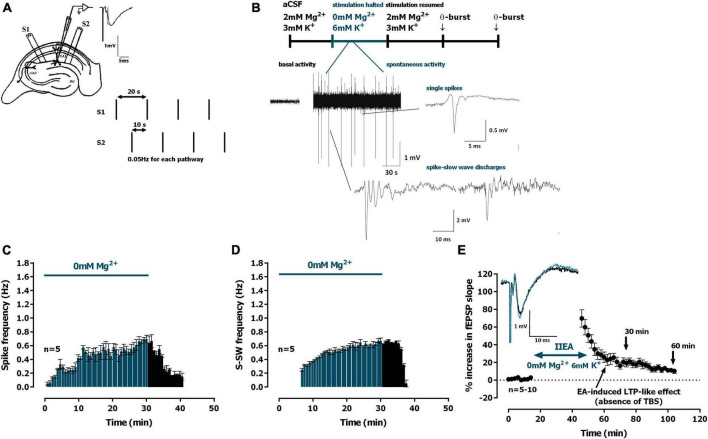
Interictal-like epileptiform activity induced by *0Mg^2+^* and long-term potentiation (LTP)-like effect. **(A)** Electrophysiological recordings of field excitatory post-synaptic potentials (fEPSPs) evoked by electrical stimulation in the CA1 area of hippocampal slices. The same recording configuration was used when monitoring spontaneous activity, but stimulation was halted during the 30 min perfusion with artificial cerebrospinal fluid (aCSF) lacking Mg^2+^ and containing 6 mM K^+^ (*0Mg^2+^*). **(B)** Time-course of a typical experiment showing the spontaneous activity recorded prior to *0Mg^2+^* arrival to the superfusion chamber (left) and during the 30 min *0Mg^2+^* perfusion. Traces of the two main types of epileptiform activity recorded, single *population spikes* and *spike-slow wave* activity, are shown at the millisecond scale. **(C,D)** Averaged time-course of the frequency of spontaneous *population spikes* **(C)** and *spike-slow wave* activity [**(D)**, *n* = 5]. **(E)** Averaged time-course of changes in fEPSP slope caused by *0Mg^2+^* perfusion. *Inset*: traces of fEPSPs obtained in the same experiment before (black) and 25–30 min after *0Mg^2+^* perfusion (blue). Electrophysiological recordings of fEPSPs evoked by electrical stimulation were performed in the CA1 area of hippocampal slices. Traces are the average of eight consecutive responses and are composed of the stimulus artifact, the presynaptic volley and the fEPSP. The mean ± S.E.M is depicted **(C,E)**.

Hippocampal slices (Aidil-Carvalho et al., 2017) were kept in a resting chamber in the same oxygenated aCSF at room temperature 22–25^°^C for at least 1 h for recovery, then one slice at a time was transferred to a submerged recording chamber of 1 ml capacity, where it was continuously superfused at a rate of 3 ml/min with the same oxygenated solution kept at 32^°^C. Stimulation (rectangular pulses of 0.1 ms, 100–260 μA) was delivered through a bipolar concentric wire electrode placed on the Schaffer collateral/commissural fibers in the *stratum radiatum.* Two separate sets of the Schaffer collaterals (S1 and S2) were stimulated alternately every 10 s, each pathway being stimulated every 20 s (0.05 Hz, Figure 1A). Extracellular recordings of field excitatory post-synaptic potentials (fEPSP) were obtained from CA1 *stratum radiatum* through micropipettes 4 M NaCl filled (2–4 MΩ resistance) using an Axoclamp-2B amplifier (10 Vm pre-amplifier, Axon Instruments, Molecular Devices, San Diego, CA, USA). fEPSPs were evoked on the two pathways, and the initial intensity of the stimulus, of comparable magnitude in both pathways, elicited a fEPSP of 600–1,000 μV amplitude (near 50% of the maximal response), yet avoiding signal contamination by the population spike. The fEPSPs signals were filtered at –3 bB with the inbuilt filter of the Axopatch 2-B, digitized at 20 kHz using a BNC-2110 (National Instruments, Austin, TX, USA) interface and acquired by a laboratory computer with the WinLTP software (Anderson and Collingridge, 2001, 2007). The averages of six consecutive evoked fEPSPs from each pathway were obtained, measured, graphically plotted, and recorded for further analysis. The fEPSPs were quantified as the slope of the initial phase of the potential.

Spontaneous epileptiform activity (EA) was induced after a stable fEPSP slope baseline was obtained for at least 20 min. Electrical stimulation was stopped during these EA induction protocols to allow for the monitoring of pure spontaneous activity. Interictal-like epileptiform activity was induced by removing Mg^2+^ from the superfusion media for 30 min (*0Mg^2+^*) while increasing the K^+^ concentration to 6 mM. These concentrations were chosen to assure the short-term onset of EA (Gloveli et al., 1995; Antonio et al., 2016). Ictal-like EA was induced by perfusion with aCSF containing bicuculline (10 μM, *Bic*) for 15 min. Electrical stimulation was resumed when spontaneous activity halted. Analysis of spontaneous activity was performed by visual inspection of 10 s windows, identification, and manual recording of spontaneous events for all signals above 200 μV amplitude.

### Influence of epileptiform activity on PPF and LTP

Altered synaptic properties following EA could involve changes in presynaptic regulation of neurotransmitter release. As such, we tested the influence of EA on PPF, a synaptic short-term synaptic plasticity property that could reveal such changes. PPF of fEPSP slope is believed to result from an enhancement in neurotransmitter release at CA3–CA1 synapses in response to the second stimulation due to the presence of residual calcium generated by the first stimulation in glutamatergic terminals (Zucker, 2003). This facilitation occurs because the release probability at these synapses is low under the current stimulation conditions (Wu and Saggau, 1994; Debanne et al., 1996). Although PPF in the CA1 area of the hippocampus has also been attributed to paired-pulse depression in the fast inhibitory postsynaptic potential (IPSP) (Nathan and Lambert, 1991), given that most synapses in the *stratum radiatum* are excitatory (Megías et al., 2001), PPF of fEPSP slope is mostly attributed to presynaptic changes in glutamate release.

To test the influence of EA on paired-pulse facilitation (PPF), PPF was elicited on the same pathway prior and 30 min after EA induced either by *0Mg^2+^* or *Bic* perfusion. The stimulation protocol used to induce PPF was the delivery of two consecutive pulses with 50 ms interpulse interval. The synaptic facilitation was quantified as the ratio P2/P1 between the slopes of the fEPSP elicited by the second P2 and the first P1 stimuli.

Long Term Potentiation (LTP) was induced by a mild theta burst stimulation (TBS) pattern (five trains of 100 Hz, four stimuli, separated by 200 ms) and was particularly chosen to avoid having a ceiling effect on LTP following EA. The TBS train used to induce LTP was delivered 30, 60, and 90 min after termination of EA induction protocols, provided a stable baseline was obtained for at least 16 min. LTP in control conditions (absence of prior EA) was time-matched to experiments performed 30 min after EA. Stimulus intensity was not altered during these stimulation protocols. LTP intensity was calculated as the % change in the average slope of the potentials taken from 50 to 60 min after TBS, compared to the average slope of the fEPSP measured during the 12 min that preceded TBS. Control and test conditions (for which epileptiform activity was induced prior to TBS) were tested in independent slices. LTP induced at different time points following EA was in some (but not all) cases tested in independent pathways on the same slice. In all experiments S1 always refers to the first pathway (left or right, randomly assigned) to which TBS was applied. Each *n* represents a single LTP experiment) performed in one slice from an independent animal, i.e., *n* denotes the number of animals.

The independence of the two pathways regarding LTP expression was tested at the end of the experiments by studying paired-pulse facilitation (PPF) across both pathways. i.e., now the two Schaffer pathways were consecutively stimulated with 50 ms interpulse interval. The synaptic facilitation was quantified as the ratio P2/P1 between the slopes of the fEPSP elicited by the second P2 and the first P1 stimuli and less than 10% facilitation was observed.

### Western blot analysis of synaptic lipid raft markers and AMPA receptor changes influencing synaptic plasticity, and GluA1 phosphorylation

Hippocampal slices were prepared as described above and allowed for functional recovery. Then four slices were introduced in placed in 100 μl Perspex chambers and superfused with oxygenated aCSF at 32^°^C at a flow rate of 3 ml/min. Field stimulation was delivered once every 15 s in the form of rectangular pulses (1 ms duration, and an amplitude of 8 V) through platinum electrodes located above and below the slices. The electrical pulses were continuously monitored with an oscilloscope. In test (but not in control) slices interictal-like and ictal EA was induced with *0Mg^2+^* and *Bic* perfusion as described above for electrophysiological experiments. Stimulation lasted for 30 min before induction of EA, it was halted during EA and was resumed for 30 min (average duration of an electrophysiological experiment until first TBS delivery), after which slices were collected. Hippocampal synaptosomes were isolated from hippocampal slices as previously described (Cunha-Reis et al., 2017). Briefly, at the end of stimulation, slices were collected in sucrose solution (320 mM Sucrose, 1 mg/ml BSA, 10 mM HEPES e 1 mM EDTA, and pH 7,4) containing protease (complete, mini, EDTA-free Protease Inhibitor Cocktail, and Sigma) and phosphatase (1 mM PMSF, 2 mM Na_3_VO_4_, and 10 mM NaF) inhibitors and homogenized with a Potter-Elvejham apparatus. Each sample (*n* = 1) was obtained from several slices (minimum four per condition) from 1 to 2 animals. The suspension was centrifuged at 3,000 g for 10 min, the supernatant collected and further centrifuged at 14,000 g for 12 min, and the pellet resuspended in 3 ml of a Percoll 45% (v/v) in modified aCSF (20 mM HEPES, 1 mM MgCl_2_, 1.2 mM NaH_2_PO_4_, 2.7 mM NaCl; 3 mM KCl, 1.2 mM CaCl_2_, 10 mM glucose, and pH 7.4). After centrifugation at 14,000 g for 2 min at 4^°^C, the top layer (synaptosome fraction) was washed twice with modified aCSF also containing protease and phosphatase inhibitors and hippocampal membranes were resuspended at a concentration of 1 mg/ml protein concentration (Bradford assay) in modified aCSF. Aliquots of this suspension of hippocampal membranes were snap-frozen in liquid nitrogen and stored at –80^°^C until Western-blot analysis. All samples were analysed in duplicate in western-blot experiments.

For western blot, samples were incubated for 5 min at 95^°^C with Laemmli buffer (125 mM Tris-BASE, 4% SDS, 50% glycerol, 0,02% Bromophenol Blue, and 10% β-mercaptoethanol), run on standard 10% sodium dodecyl sulphate polyacrylamide gel electrophoresis (SDS-PAGE) and transferred to PVDF membranes (Immobilon-P transfer membrane PVDF, pore size 0.45 μm, Immobilon) and blocked for 1 h with either 3% BSA or 5% milk (Caulino-Rocha et al., 2022). Membranes were incubated overnight at 4^°^C with mouse anti-gephyrin (#147011, Synaptic Systems, AB_2810214), rabbit anti-PSD-95 (#CST-2507, Cell Signaling Tech., AB_561221), mouse anti-caveolin-1 (#ab106642, Abcam, AB_10861399), mouse anti-flotillin-1 (#ab133497, Abcam, AB _11156367), rabbit antiphospho-Ser845-GluA1 (1:2500, Abcam #Ab76321; RRID:AB_1523688), rabbit antiphospho-Ser-831-GluA1 (1:2000, Abcam #Ab109464; RRID:AB_10862154), rabbit anti-GluA1 (1:4000, Millipore #AB1504; RRID:AB_2113602), rabbit anti-GluA2 (1:1000, Proteintech #11994-1-AP; RRID:AB_2113725), rabbit anti-alpha-tubulin (1:5000, Proteintech #11224-1-AP; RRID:AB_2210206) and rabbit anti-beta-actin (1:10000, Proteintech, Cat# 60008-1; RRID:AB_2289225) primary antibodies. After washing the membranes were incubated for 1 h with anti-rabbit or anti-mouse IgG secondary antibody both conjugated with horseradish peroxidase (HRP) (Proteintech) at room temperature. HRP activity was visualized by enhanced chemiluminescence with ECL Plus Western Blotting Detection System using a ImageQuant™ LAS imager (GE Healthcare, Portugal). Band intensity was estimated using the ImageJ software. β-actin or α-tubulin band density was taken as a loading control and used for data normalization. The % phosphorylation for each AMPA GluA1 subunit target was calculated normalizing the change in phosphorylated form band intensity by the change in band intensity of the total GluA1 immunostaining.

### Materials

Bicuculline methochloride (Ascent Scientific, UK) was made up in 10 mM stock solutions in DMSO. Aliquots of the stock solutions were kept frozen at –20^°^C until use. An aliquot was diluted in aCSF for use in each experiment. The maximal DMSO concentration used (0.01% v/v, *n* = 4) was devoid of effects on fEPSP slope.

### Data and statistical analysis

Long term potentiation (LTP) values are depicted as the mean ± S.E.M of *n* experiments. Each *n* represents a single experiment performed in slices obtained from one different animal for LTP experiments (i.e., *n* stands for the number of animals). For western blot experiments each sample was obtained from slices from 1 to 2 animals (i.e., each *n* stands for one or two animals combined). Statistical analysis was performed using GraphPad Prism 6.01 for Windows. In electrophysiology experiments, significance of the means of LTP-like effects was evaluated using Student’s *t*-test. Significance of the differences between the LTP means was evaluated using one-way ANOVA with Sidak *post-hoc* test. Repeated measures ANOVA with Sidak’s *post-hoc* test (when F was significant) was used to evaluate group differences in western blot experiments. No outliers were identified in our data (ROUT method).

## Results

To understand the impact of EA on the function of hippocampal synapses we studied its immediate influence on synaptic transmission in the CA1 area and early hour synaptic plasticity using electrophysiological recordings. We characterized spontaneous activity during seizure-inducing conditions, changes in electrically evoked synaptic transmission and LTP induced by mild TBS after EA.

### Influence of interictal-like EA on basal synaptic transmission and LTP expression

The evoked fEPSPs recorded under basal stimulation conditions in the *stratum radiatum* of the CA1 area in hippocampal slices from young-adult rats (Figure 1A) had an average slope of 0.635 ± 0.026 mV/ms (*n* = 26), that represented 40–60% of the maximal response in each slice. Upon superfusion with modified aCSF lacking Mg^2+^ (*0Mg^2+^*) for 30 min in the absence of electrical stimulation spontaneous activity started gradually with single spontaneous population spikes (Figure 1B, 0.7 to 1.2 mV amplitude, *n* = 5) and 6–8 min later larger amplitude (2.7 to 4 mV, *n* = 5) population spikes with wave reverberation (spike-slow wave discharges, *S-SW*, Figure 1B) were observed, as previously described (Gloveli et al., 1995). The maximum frequencies were reached near the end of the 30 min superfusion with *0Mg^2+^* and were of 0.695 ± 0.055 *spikes*/min (*n* = 5, Figure 1C) and of 0.670 ± 0.040 *S-SW*/min (*n* = 5, Figure 1D). All spontaneous activity was terminated within 8–12 min post *0Mg^2+^* washout. When resuming electrical stimulation an *LTP-like effect* was observed in the slope of the evoked fEPSPs (Figure 1E) with a remaining potentiation of 21.0 ± 4.7% (*n* = 10) 30 min after resuming electrical stimulation and of 10.7 ± 1.8% (*n* = 5) at 60 min.

**FIGURE 2:**
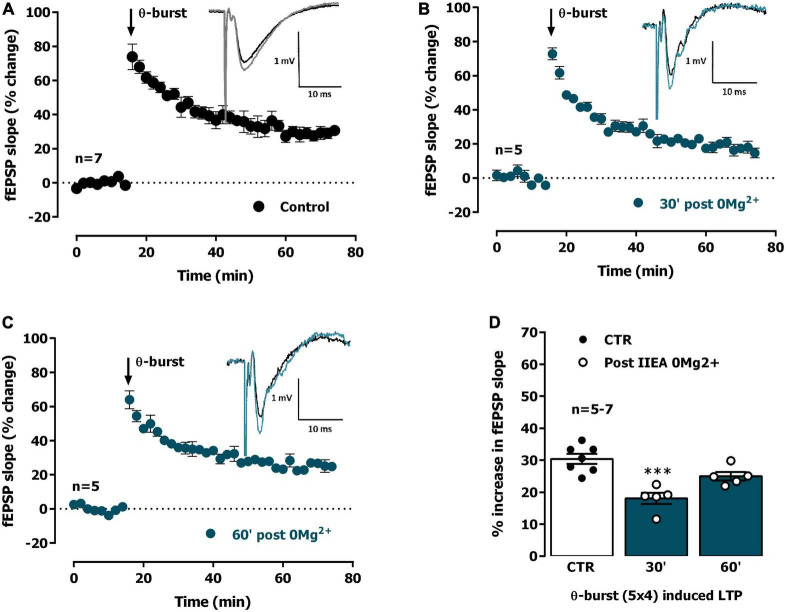
Interictal-like activity transiently impairs the expression of hippocampal CA1 long-term potentiation (LTP) of synaptic transmission. **(A)** Averaged time-course of changes in field excitatory post-synaptic potentials (fEPSP) slope (-●-) caused by theta-burst stimulation (TBS, five bursts at 5 Hz, each composed of four pulses at 100 Hz) in experiments in which a control slice was stimulated in the absence of added drugs. *Inset:* Traces of fEPSPs obtained in one of the same experiments before (black) and 50–60 min after TBS (gray). Traces are the average of eight consecutive responses and are composed of the stimulus artifact, the presynaptic volley and the fEPSP. **(B,C)** Averaged time-course of changes in fEPSP slope caused by TBS applied either 30 min **(B)** or 60 min **(C)** after interictal epileptiform activity induced by *0Mg^2+^* perfusion (-●-). **(D)** LTP magnitude estimated from the averaged enhancement of fEPSP slope observed 50–60 min after TBS in control slices (●, left open bar) or in slices previously experiencing interictal epileptiform activity induced by *0Mg^2+^* (°, blue bars). LTP was induced either 30 min (middle bar) or 60 min after epileptiform activity (right bar). *Inset:* Traces of fEPSPs obtained before (black) and 50–60 min after TBS (blue). Individual values and the mean ± S.E.M are depicted **(D)**. ***p < 0.001 (one-way ANOVA) as compared to LTP magnitude in control slices (°, in the left).

We thus studied the influence of *0Mg^2+^*-induced EA on LTP expression. When mild TBS was applied in control conditions the LTP magnitude corresponded to a 32.0 ± 1.3% enhancement of fEPSP slope 50–60 min after TBS (*n* = 13, Figure 2A), an effect that was absent when slices were stimulated in the presence of the NMDA receptor antagonist AP-5 (100 μM), as previously described (Rodrigues et al., 2021). When a TBS train was delivered 30 min past interictal-like EA induced by *0Mg^2+^*, LTP expression was impaired (% increase in fEPSP slope: 18.0 ± 1.8%, *n* = 5, Figure 2B). When delivered to the slices 60 min past interictal-like EA, the same TBS stimulation elicited a larger LTP, nearly as big as the one obtained in control slices, now increasing by 24.8 ± 1.1% (*n* = 5) the fEPSP slope 50–60 min post TBS (Figure 2C). This suggests interictal-like EA induced by *0Mg^2+^* does not promote marked or sustained changes in the LTP expressing ability of CA3 to CA1 synapses, that are only transiently affected by this type of EA.

### Influence of ictal-like EA on basal synaptic transmission and LTP expression

**FIGURE 3:**
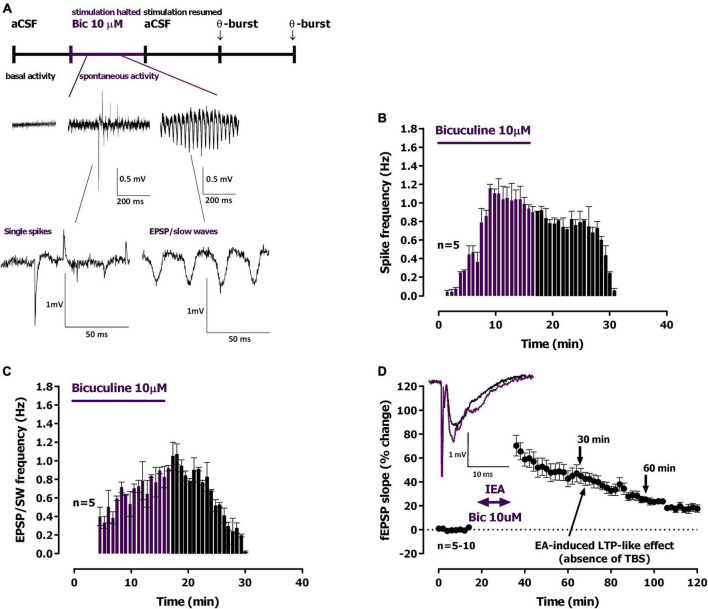
Ictal-like epileptiform activity induced by *Bicuculline* and long-term potentiation (LTP)-like effect. **(A)** Time-course of a typical experiment showing the spontaneous activity recorded prior to bicuculline (10 μM, *Bic*) delivery to the superfusion chamber (left) and during the 16 min *Bic* perfusion. Specific examples of the two main types of epileptiform activity recorded, single *population spikes* and *EPSP-slow wave* activity, are shown at the millisecond scale. Stimulation was halted during *Bic* perfusion. **(B**,**C)** Averaged time-course of the frequency of spontaneous *population spikes* **(B)** and *EPSP-slow wave* activity [**(C)**, *n* = 5]. **(D)** Averaged time-course of changes in eld excitatory post-synaptic potentials (fEPSP) slope caused by *Bic* perfusion. *Inset*: traces of fEPSPs obtained in the same experiment before (black) and 25–30 min after *Bic* perfusion (purple). Electrophysiological recordings of fEPSPs evoked by electrical stimulation were performed in the CA1 area of hippocampal slices. Traces are the average of eight consecutive responses and are composed of the stimulus artifact, the presynaptic volley and the fEPSP. The mean ± S.E.M is depicted **(B,D)**.

Upon superfusion with modified aCSF containing bicuculline (10 μM, *Bic*) for 15 min spontaneous activity started with single isolated population spikes (Figure 3A, 0.51 to 0.89 mV amplitude, *n* = 7) that were increasingly more frequent, and more than the ones observed during *0Mg^2+^* perfusion (Figure 3B). Within 4–6 min of *Bic* superfusion slower waves of synchronous activity (0.29 to 0.68 mV, *n* = 7, Figure 3C) began to develop showing occasional (0.5 to 1 per min) high amplitude bursts (EPSP / slow-wave discharges, *EPSP-SW*, Figures 3A, C). The maximum EPSP-SW frequencies were reached by the end the 16 min superfusion with *Bic* and were of 1.070 ± 0.110 *S-SW*/min (*n* = 5, Figure 3C). The maximum spike frequencies were reached shortly (6–8 min) after beginning the 16 min superfusion with *Bic* and were of 1.040 ± 0.140 *spikes*/min (*n* = 5, Figure 3B). All spontaneous activity was terminated within 16–20 min of *Bic* washout. When resuming electrical stimulation an *LTP-like effect* was observed causing a potentiation of 45.1 ± 6.2% (*n* = 10) in the slope of the evoked fEPSPs (Figure 3D) obtained 30 min after resuming electrical stimulation, of 25.3 ± 2.5% (*n* = 7) at 60 min and of 14.5 ± 1.3% (*n* = 5) at 90 min.

**FIGURE 4:**
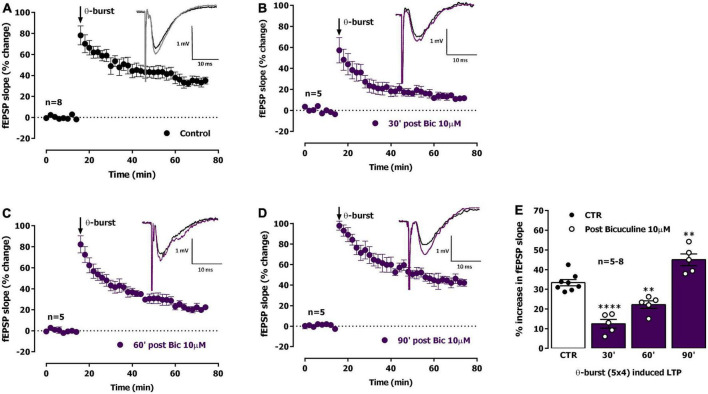
Ictal-like activity induces a biphasic alteration in the expression of hippocampal CA1 long-term potentiation (LTP) of synaptic transmission. **(A)** Averaged time-course of changes in fEPSP slope (-●-) caused by theta-burst stimulation (TBS, five bursts at 5 Hz, each composed of four pulses at 100 Hz) in experiments in which a control slice was stimulated in the absence of added drugs. *Inset:* Traces of field excitatory post-synaptic potentials (fEPSPs) obtained in one of the same experiments before (black) and 50–60 min after TBS (gray). Traces are the average of eight consecutive responses and are composed of the stimulus artifact, the presynaptic volley and the fEPSP. **(B–D)** Averaged time-course of changes in fEPSP slope caused by TBS delivered either 30 min **(B)**, 60 min **(C)** or 90 min **(D)** after ictal epileptiform activity induced by bicuculline 10 μM (*Bic*) perfusion (-●-). **(E)** LTP magnitude estimated from the averaged enhancement of fEPSP slope observed 50–60 min after TBS in control slices (●, left open bar) or in slices previously experiencing interictal epileptiform activity induced by *Bic* (°, purple bar). LTP was induced either 30 min (second bar), 60 min (third bar) or 90 min (last bar) after epileptiform activity. *Inset:* Traces of fEPSPs obtained before (black) and 50–60 min after TBS (purple). The mean ± S.E.M is depicted **(A–D)**. **p < 0.01 and **** *p* < 0.0001 (one-way ANOVA) as compared to LTP magnitude in control slices (°, in the left).

We thus studied the influence of *Bic*-induced EA on LTP expression. LTP induced by mild TBS 30 min following ictal-like EA elicited by *Bic* was strongly impaired (% increase in fEPSP slope: 12.5 ± 2.2%, *n* = 5, Figure 4B). TBS stimulation delivered to the slices 60 min past ictal-like EA, still elicited an impaired LTP, i.e., smaller than the one obtained in control slices, now increasing by 22.2 ± 1.9% (*n* = 5) the fEPSP slope 50–60 min post TBS (Figure 4C). When delivered to the slices 90 min past ictal-like EA, TBS stimulation now induced an LTP larger than the one obtained in control slices, thus increasing by 45.0 ± 3.0% (*n* = 5) the fEPSP slope 50–60 min post TBS (Figure 4D). As such, a biphasic change in the ability of TBS to induce LTP at CA1 hippocampal synapses occurs following ictal-like EA, first reflecting an impairment, then a mildly enhanced capacity to elicit LTP by TBS. This is likely an overshoot caused by recovery mechanisms (Russo et al., 2013).

### Influence of EA on PPF

Altered LTP expression following EA could be due to changes in presynaptic regulation of neurotransmitter release. As such, we also investigated the influence of EA on PPF of the fEPSP slope, a synaptic short-term synaptic plasticity property of CA3–CA1 synapses, where the release probability upon basal stimulation is low, that results from an enhancement in neurotransmitter release in response to the second stimulation due to the presence of residual calcium generated by the first stimulation (Wu and Saggau, 1994; Debanne et al., 1996). PPF of fEPSP slope is mostly due to presynaptic changes in glutamate release.

**FIGURE 5:**
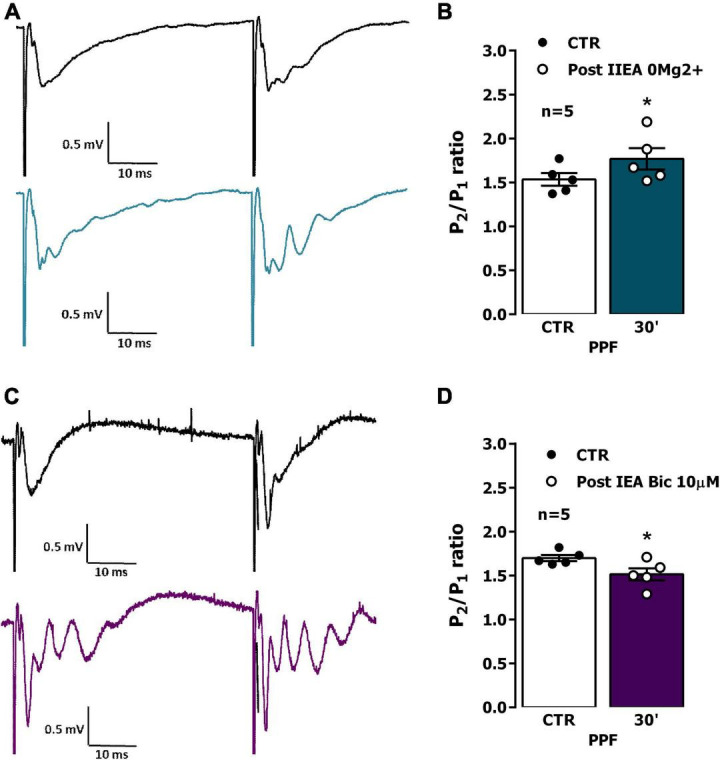
Impact of epileptiform activity on paired-pulse facilitation (PPF) of synaptic transmission in the CA1 area of the hippocampus. PPF was induced by applying two consecutive stimuli with 50 ms interval once every 20 s while recording field excitatory post-synaptic potentials (fEPSPs). **(A,C)** Representative recordings of fEPSPs pairs obtained when using the PPF paradigm before (top) and 30 min after interictal epileptiform activity induced by *0Mg^2+^* **(A)**, bottom, blue line or ictal epileptiform activity induced by *Bic* **(C)** bottom, purple line. Traces are the average of eight consecutive responses and are composed of the stimulus artifact, the presynaptic volley and the fEPSP. Influence of interictal epileptiform activity induced by *0Mg^2+^* **(B)** or of ictal epileptiform activity induced by *Bic* **(D)** on PPF magnitude estimated from change in the fEPSP slope ratio (S_2_/S_1_) between responses evoked by the first (conditioning) and the second (test) stimulation. In **(B,D)**, each bar represents the mean ± S.E.M of results obtained in five experiments. *P < 0.05 (paired Student’s *t*-test) as compared with S_2_/S_1_ ratio prior to induction of epileptiform activity.

When two consecutive stimulation pulses were delivered with a 50 ms interval to the Schaffer collateral/commissural pathway in the CA1 area, the slope of the fEPSP elicited by the second (test, S2) stimulation pulse was 1.4–1.8 times higher than the one evoked by the first (conditioning, S1) stimulation pulse (Figure 5A). Facilitation of glutamatergic transmission may play together with depression of GABAergic transmission in promoting PPF of fEPSP slope in hippocampal CA1. Most synapses in the *stratum radiatum* are excitatory (Megías et al., 2001), as such, changes in PPF of fEPSP slope are most likely predominantly due to presynaptic changes in glutamate release. Following interictal EA induced by *0Mg^2+^* PPF was enhanced (% increase in S_2_/S_1_ ratio was 15.0 ± 3.7% *n* = 5, *P* < 0.05, Figures 5A, B), suggesting that increased glutamate release probability is involved in the enhancement of synaptic transmission observed 30 min post interictal activity induced by *0Mg^2+^* in the CA1 area of the hippocampus. Conversely, after ictal activity induced by *Bic*, PPF was mildly reduced (% decrease in S_2_/S_1_ ratio was 11.0 ± 2.9% *n* = 5, *P* < 0.05, Figures 5C, D).

To further understand the molecular alterations at synapses that were underlying these changes in LTP expression we performed parallel experiments where hippocampal slices were also subjected to EA induced by *0Mg^2+^* or *Bic*, and 30 min later were processed for synaptosome isolation and analysis of synaptic proteins associated with altered synaptic plasticity. These included AMPA receptor subunits GluA1 and GluA2, GluA1 phosphorylation in Ser831 and Ser845, Kv4.2 potassium channels, synaptic structural and lipid raft markers such as PSD-95, gephyrin, caveolin-1, flotillin-1, α-tubulin, and synaptophysin-1.

### Influence of interictal-like and ictal-like EA on AMPA receptor composition and phosphorylation supporting LTP expression

**FIGURE 6:**
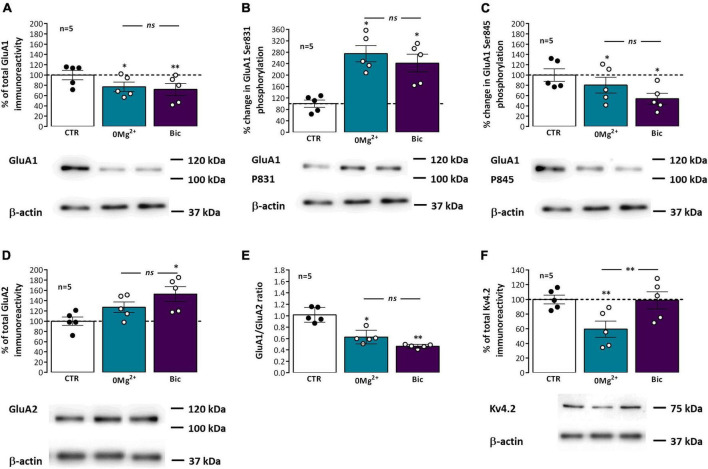
Impact of epileptiform activity on phosphorylation of synaptic hippocampal AMPA GluA1 subunits on Ser 831 and Ser 845, GluA1/GluA2 ratio and Kv4.2 levels. Each panel shows at the bottom the western-blot immunodetection of AMPA total GluA1 subunits **(A)**, its phosphorylated forms in Ser831 **(B)** and Ser845 **(C)**, total GluA2 subunits **(D)** and Kv4.2 potassium channels **(F)** obtained in one individual experiment where hippocampal slices underwent Schaffer collateral basal stimulation for 20 min and then were subjected either to interictal-like epileptiform activity (EA) induced by perfusion with *0Mg^2+^* for 30 min or ictal-like EA by exposure to bicuculine (10 μM, *Bic*) for 16 min, and then allowed to recover for 30 min before slice collection. Control slices were monitored for 70 min (the equivalent time of the EA protocol) before WB analysis. Western blot experiments were performed using synaptosome preparations obtained from these slices. Respective total GluA1 immunoreactivity **(A)**, % GluA1 phosphorylation in Ser831 **(B)** or Ser845 **(C)**, residues, total GluA2 immunoreactivity **(D)**, GluA1/GluA2 ratios **(E)** and total Kv4.2 potassium channel levels **(F)** are also plotted at the top for each panel. Individual values and the mean ± S.E.M of five independent experiments are depicted. 100% - averaged GluA1 or GluA2 immunoreactivity or GluA1 phosphorylation obtained in control conditions (CTR, absence of EA). *P < 0.05 and **P < 0.01 (*ANOVA*, Sidak’s multiple comparison test) as compared to CTR; *ns* represents non -significant differences *P* > 0.05 (*ANOVA*, Sidak’s multiple comparison test) between respective bars.

AMPA receptor subunit composition (GluA1-4) influences channel function and LTP outcomes (Chater and Goda, 2022), since AMPA receptors lacking GluA2 are Ca^2+^ permeable and when activated can further add to the postsynaptic Ca^2+^ rise and LTP levels. To elucidate the possible changes in synaptic AMPA receptor subunit composition that may impact its Ca^2+^ permeability and its contribution to altered LTP expression following EA we investigated changes in synaptic GluA1 and GluA2 levels and GluA1/GluA2 ratio. Following EA, the synaptic levels of AMPA GluA1 subunits (Figure 6A) were decreased to 77.1 ± 9.0% (*n* = 5) after *0Mg^2+^* exposure and to 72.0 ± 11.8% (*n* = 5) after *Bic* exposure. Opposingly, synaptic GluA2 subunits (Figure 6D) were increased to 127.3 ± 10.1% (*n* = 5) after *0Mg^2+^* exposure and to 152.8 ± 14.3% (*n* = 5) after *Bic* exposure. Consequently, GluA1/GluA2 ratios (Figure 6E) were also markedly reduced after *0Mg^2+^* and *Bic* exposure. No significant differences were found between *0Mg^2+^* or *Bic* exposure for both targets as well as GluA1/GluA2 ratios.

Long term potentiation (LTP) expression, and particularly mild TBS induced LTP, depends on the Ca^2+^-dependent auto-phosphorylation of Ca^2+^/calmodulin dependent protein kinase II (CaMKII), that influences the phosphorylation of AMPA GluA1 subunits and their synaptic recruitment (Appleby et al., 2011; Rodrigues et al., 2021). These are also targeted by other intracellular kinases like protein kinase A and C (PKA and PKC) affecting hippocampal LTP expression by supporting traffic or altering AMPA receptor opening probability (Lee et al., 2003). Although not directly involved in mild TBS-induced LTP (Rodrigues et al., 2021), altered PKA and PKC activity during EA could influence AMPA basal phosphorylation thus influencing subsequent TBS-induced LTP. We thus investigated if altered GluA1 phosphorylation levels at Ser831, a prominent target of CaMKII and PKC, and Ser845, a main target of PKA were implicated in altered LTP responses following EA. Phosphorylation of GluA1 subunits in Ser831 (Figure 6B) was markedly increased to 275.0 ± 12.7% (*n* = 5) after *0Mg^2+^* induced EA and 241.9 ± 31.1% (*n* = 5) after *Bic* induced EA. Opposingly, phosphorylation of GluA1 subunits in Ser845 (Figure 6C) was decreased to 80.4 ± 15.4% (*n* = 5) after *0Mg^2+^* exposure and 53.9 ± 10.6% (*n* = 5) after *Bic* exposure. No significant differences were found between *0Mg^2+^* or *Bic* exposure for the phosphorylation on both targets.

Kv4.2 K^+^ channels are important regulators of dendritic excitability by mediating the transient A-current. Its membrane levels and phosphorylation status are regulated by electrical activity patterns used in LTP induction (e.g., strong TBS), suggesting that the activity of the channel, contributes to LTP expression (Frick et al., 2004; Kim and Hoffman, 2008; Rodrigues et al., 2021). Following EA, Kv4.2 levels (Figure 6F) were reduced to 59.5 ± 11.1% (*n* = 5) after *0Mg^2+^* exposure but were not significantly altered (98.8 ± 11.7%, *n* = 5) after *Bic* induced EA.

### Influence of EA on lipid raft associated proteins

**FIGURE 7:**
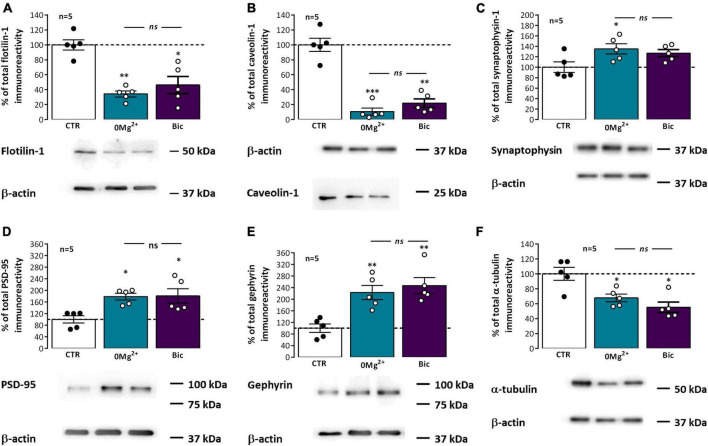
Impact of epileptiform activity on classic and synaptic lipid raft markers. Each panel shows at the bottom the western-blot immunodetection of flotillin-1 **(A)**, caveolin-1 **(B)**, synaptophysin-1 **(C)**, PSD-95 **(D)**, gephyrin **(E)**, and alpha-tubulin **(F)** obtained in one individual experiment where hippocampal slices underwent Schaffer collateral basal stimulation for 20 min and then were subjected either to interictal-like epileptiform activity (EA) induced by perfusion with *0Mg^2+^* for 30 min or ictal-like EA by exposure to bicuculline (10 μM, *Bic*) for 16 min, and then allowed to recover for 30 min before slice collection. Control slices were monitored for 70 min (the equivalent time of the EA protocol) before WB analysis. Western blot experiments were performed using synaptosome preparations obtained from these slices. Respective average change in total flotillin-1 **(A)**, caveolin-1 **(B)**, synaptophysin-1 **(C)**, PSD-95 **(D)**, gephyrin **(E)**, and alpha-tubulin **(F)** immunoreactivities are also plotted at the top in each panel. Individual values and the mean ± S.E.M of five independent experiments are depicted. 100% - averaged PSD-95, gephyrin, caveolin-1, flotillin-1, or synaptophysin-1 immunoreactivity in control conditions (CTR, absence of EA). *P < 0.05, **P < 0.01, and ***P < 0.001 (*ANOVA*, Sidak’s multiple comparison test) as compared to CTR; *ns* represents non-significant differences *P* > 0.05 (*ANOVA*, Sidak’s multiple comparison test) between respective bars.

Pre and postsynaptic membrane lipids differ substantially from the ones in the remaining neuronal membrane, hinting that synaptic signaling is strongly dependent on this specialization (Borroni et al., 2016; Flores et al., 2019) and different classes of membrane lipids integrate domains showing distinctive properties in terms of size, rigidity, and thickness (Wilson et al., 2020). Lipid rafts, liquid-ordered or gel-like membrane nanodomains segregated from the bulk membrane and enriched in cholesterol, sphingolipids and GPI-anchored proteins, are crucial for dynamic synaptic signaling. These comprise two main types of structure, caveolae, curved membrane domains, or planar lipid rafts (Allen et al., 2007; Borroni et al., 2016), characterized by the presence of the membrane proteins caveolins, a family of 22 kDa cholesterol-binding membrane proteins, and flotillins, a family of 49 kDa membrane proteins associated with the membrane inner leaflet, respectively. These play also an active role in promoting the phase separation within the membrane that maintains these lipid nanodomains at synapses (Allen et al., 2007; Frick et al., 2007). Synaptic lipid rafts can also be associated with synaptic lipid-raft nucleating/associated proteins like PSD-95 or gephyrin. Following EA, the synaptic levels of flotillin-1 (Figure 7A) were markedly reduced to 34.3 ± 4.1% (*n* = 5) after *0Mg^2+^* exposure and 46.3 ± 11.4% (*n* = 5) after *Bic* exposure while the levels of caveolin-1 (Figure 7B) were dramatically reduced to 10.6 ± 4.7% (*n* = 5) after *0Mg^2+^* exposure and 21.9 ± 5.6% (*n* = 5) after *Bic* exposure. Regarding the postsynaptic scaffolding protein of glutamatergic synapses PSD-95, after EA, it was observed an increase in PSD-95 levels to 178.6 ± 11.5% (*n* = 5) following *0Mg^2+^* exposure and to 180.8 ± 24.9% (*n* = 5) upon *Bic* exposure (Figure 7D). Gephyrin was also significantly altered 30 min next to EA, increasing to 222.9 ± 24.6% (*n* = 5) upon *0Mg^2+^* exposure and 246.8 ± 27.8% (*n* = 5) upon *Bic* exposure (Figure 7E). Only for PSD-95 was observed a significant difference in the EA response between *0Mg^2+^* and *Bic* exposure.

To understand the relation between the changes in these different proteins at synapses and overall synaptic alterations in this synaptosomal preparation we also evaluated the synaptic levels of synaptophysin-1, a synaptic vesicle associated glycoprotein. Synaptophysin-1 levels were mildly enhanced following exposure to both *0Mg^2+^* (135.1 ± 10.0%, *n* = 5) or *Bic* (126.9 ± 6.7%, *n* = 5). In addition, we investigated changes in the structural protein α-tubulin, since our preliminary studies using it as a western blot loading control suggested that the synaptic levels of this protein were also significantly changing after EA (Figure 7F). The synaptic levels of α-tubulin were decreased to 67.7 ± 5.1% (*n* = 5) following *0Mg^2+^* exposure and 55.0 ± 7.0% (*n* = 5) after *Bic* exposure.

## Discussion

In the present work we first describe that: (1) interictal-like EA induces a mild impairment in TBS-induced LTP observed 30 min after the insult that is fully recovered 1 h after; (2) ictal-like EA, in turn, induces a marked impairment in TBS-induced LTP within 30 min -1 h after the insult and this is reversed to a mild enhancement 1 h 30 m after; (3) Impairment of LTP induced by EA was globally associated to a decrease in AMPA GluA1 subunit levels and an increase in GluA2 subunit levels, resulting in a marked decrease of the GluA1/GluA2 ratio, together with a marked increase in GluA1 P831 phosphorylation and a mild decrease in GluA1 P845 phosphorylation; (4) Kv4.2 K^+^ channels levels were decreased following interictal-like EA but not following ictal-like EA; (5) EA also decreased the levels of classic lipid raft markers caveolin-1 and flotillin-1, while increasing synapse specific lipid raft markers PSD-95 and, most prominently, gephyrin.

In this work, the observed physiological alterations in LTP induction and expression, together with the alterations in the molecular composition of synapses, provide a good insight on the very early alterations of synaptic plasticity in adult rodents following two forms of epileptiform activity: (1) seizure-like activity (the one induced by bicuculline, 10 μM, *Bic*) and (2) neuronal interictal-like activity (induced by Mg^2+^ suppression, *0Mg^2+^*), characteristic resting brain activity pattern of epilepsy patients, often observed in the latent period of epileptogenesis in animal models. The fact that LTP expression is influenced by previous neuronal and/or synaptic activity is not itself new (Abraham and Bear, 1996; Colgin et al., 2004; Zhang et al., 2005) and previous studies reported structural alterations in synapse architecture following seizures (Leite et al., 2005; Postnikova et al., 2017). Altered synaptic plasticity was also observed following numerous physiologic stress conditions, (Félix-Oliveira et al., 2014; Arias-Cavieres et al., 2021) and associated with the clustering of synaptic markers and altered synaptic morphology (Sebastian et al., 2013). Nonetheless, LTP changes occurring following *in vitro* events resembling seizure-like activity versus the ones caused by altered resting neuronal activity observed in epileptic patients was never investigated concomitantly in the same model. To this purpose, the use of the hippocampal slice *in vitro* system for seizure generation was preferred to the *in vivo* model of epilepsy also used by our research group (Serpa et al., 2022), since it allowed to assess LTP in the first hours following EA, thus avoiding the dissection and recovery time after slice preparation (usually 1 h– 1 h 30 m) that would expand the time window for LTP inspection. This is of particular interest to the development of early intervention therapies to prevent epileptogenesis.

Interictal-like EA, lasting for about 30 min, was characterized by spaced large amplitude burst of neuronal activity separated by more silent periods with occasional single spikes while ictal like activity, lasting for 20–25 min, was characterized by a continuous activity showing both population spikes and EPSP-SWs. As previously observed by many others (Gloveli et al., 1995; Debanne et al., 2006; Postnikova et al., 2020; Ergina et al., 2021), synaptic transmission was potentiated following both ictal-like and interictal-like EA (Figures 1E, 3D), an LTP-like effect of EA that is similarly, dependent on NMDA receptor activation (Ben-Ari, 2001; Debanne et al., 2006). However, in this paper, we focused on the ability of EA to condition further LTP induction by mild TBS (five trains of four pulses delivered at 100 Hz with 200 ms interburst interval) in a time window from 30 min to 1 h 30 min post EA. Decreased LTP observed when induced 30 min following interictal-like EA (Figure 2), was virtually gone when LTP was induced 1 h after EA. In contrast, LTP impairment was much stronger when LTP was induced 30 min following ictal-like activity (Figure 4), and a milder impairment was still observed when LTP was induced 1 h following ictal-like EA. LTP induced 1 h 30 min after ictal-like activity was, however, slightly enhanced, suggesting an overshoot in synaptic recovery mechanisms (Russo et al., 2013). Previous studies addressed the alterations in LTP following *in vitro* 4-AP induced ictal-like EA in hippocampal slices or 3 h after Li^2+^-pilocarpine-induced seizures in juvenile rats (Kryukov et al., 2016; Postnikova et al., 2020) and report a similar small reduction of LTP. Although this partially agrees with our observations, a slightly stronger TBS pattern was used, likely a requirement to induce a robust LTP in the juvenile rats (Rodrigues et al., 2021). LTP impairment after ictal-like EA was more pronounced in our work and was nearly recovered 1 h 30 m later, likely reflecting the shorter duration of the ictal-like EA in our work. The differences in the age, the LTP and EA induction methods and/or in the time frame of LTP evaluation after EA preclude a more elaborated discussion of all these findings. In this work, ictal-like activity, although shorter in duration than interictal-like activity, had a much greater impact on hippocampal LTP. This is in line with previous theories arguing that interictal-like activity is protective in epileptogenesis (Avoli, 2001; Avoli et al., 2006) and suggests that altered LTP mechanisms are not only important for epileptogenesis but also a relevant therapeutic target in its prevention (Albensi et al., 2004, 2007; Avanzini et al., 2014).

As previously mentioned, altered LTP expression following EA could be due to changes in presynaptic regulation of glutamate release, that could be assessed studying by PPF a synaptic short-term synaptic plasticity property that is influenced by such changes. In this work, PPF was mildly increased following *0Mg^2+^* induced EA and was slightly decreased following *Bic*-induced EA, suggesting that each of the two types of EA have a different impact on presynaptic function. The reduction in PPF caused by *Bic*-induced EA, although not very pronounced, is consistent with an increased glutamate release probability and may reflect enhanced presynaptic Ca^2+^ post EA (Kovac et al., 2017). Conversely, the mild increase in PPF caused by *0Mg^2+^*-induced EA is consistent with a decrease in glutamate release probability and with a putative neuroprotective role of this type of activity in epileptogenesis. Regarding its impacts on LTP expression following EA, it can be argued that enhanced glutamate release probability following ictal-like *Bic*-induced EA may somewhat contribute to enhanced LTP impairment by promoting glutamate depletion at CA3-CA1 synapses. However, it is likely that this is only a small contribution to this effect, given the small alteration in the PPF ratio.

Induction of CA1 LTP classically relies on the NMDA-mediated postsynaptic Ca^2+^ rise (Bliss et al., 2018), that follows primary AMPA receptor mediated postsynaptic depolarization. Yet, LTP outcomes are also dependent on AMPA receptor subunit composition, a variable tetrameric combination of GluA1-4 subunits influencing channel function (Wiltgen et al., 2010; Chater and Goda, 2022). In hippocampal synapses, GluA1/2 heterodimers are the most common, although GluA2/3 heterodimers are also present (Chater and Goda, 2022). Importantly, GluA2 containing AMPA Rs, being impermeable to Ca^2+^, limit the contribution of AMPA Rs to the postsynaptic Ca^2+^ rise. Diminished levels of GluA2 containing AMPA receptors were linked to aging and pathological conditions such as brain lesions (Chater and Goda, 2022). In this work we found that synaptic GluA1 levels were decreased while GluA2 levels were increased in hippocampal slices exposed to EA (Figures 5A, D), an effect more pronounced following ictal-like activity. A concomitant decrease in GluA1/GluA2 ratio was observed for both conditions (Figure 6E). These findings suggest that epileptiform activity transiently reduces the number of Ca^2+^-permeable AMPA receptors at hippocampal synapses in adult rats, a likely endogenous neuroprotective measure. Interestingly, decreased AMPA Ca^2+^ permeability following *0Mg^2+^* induced EA was previously reported in mixed neuroglial cultures (Gaidin et al., 2019), which is consistent with our results. A recent work also implicates synaptic GluA2-lacking Ca^2+^ permeable receptors as the main event in the LTP-like effects of 4-AP induced EA in CA1 pyramidal neurons in juvenile rats (Postnikova et al., 2020). Given the different role played by AMPA subunits in synaptic plasticity at these young ages (Liu et al., 2020; Chater and Goda, 2022), it is likely that the AMPA glutamatergic component of this LTP-like effect may be distinct in adult circuitry (Goodkin et al., 2008; Avoli et al., 2022; Lévesque et al., 2022). Since the initiation of EA is itself dependent on GABAergic transmission (Avoli et al., 2022), the role of GABAergic transmission in the LTP-like effects of EA should also be elucidated. This would require a distinct approach to induce ictal-like activity than the one used in this study since direct effects of bicuculline on GABAergic receptors (Tang et al., 2023) may alter also the long-lasting response to EA.

The experimental approach used in this paper to monitor changes in synaptic AMPA receptors does not provide sufficient detail on their subsynaptic distribution (Chater and Goda, 2022), and the distinction between synaptic and perisynaptic AMPA receptors, or between pre- or postsynaptic receptors. In the context of seizure induced epileptogenesis, activation of extrasynaptic receptors by glutamate spillover from active synapses may be crucial to the establishment of metaplastic events contributing to maladaptive homeostatic plasticity such as changes in AMPA/NMDA ratio at glutamatergic synapses (Postnikova et al., 2020; Ergina et al., 2021; Chater and Goda, 2022). Future works should aim the elucidation of the exact changes occurring at synaptic and perisynaptic to clarify the contribution of AMPA receptors to early alterations in synaptic plasticity leading to epileptogenesis.

Long term potentiation (LTP) expression involves the phosphorylation of AMPA GluA1 subunits and their synaptic recruitment (Appleby et al., 2011; Park et al., 2021), a mechanism dependent on CamKII and intracellular kinases like PKA and PKC (Lee et al., 2003). Epileptiform activity could limit hippocampal LTP by inducing an activity-dependent enhancement in GluA1 phosphorylation status, thus generating a ceiling effect for LTP induction. In this work, the phosphorylation of AMPA GluA1 subunits at Ser831, a prominent target of CaMKII and PKC, was enhanced after both ictal-like and interictal like EA, an effect slightly more pronounced following interictal-like EA (Figure 6B). This suggests enhanced GluA1 phosphorylation at Ser831 contributes to the LTP-like effects of EA, likely by increasing channel conductance and promoting the recruitment of AMPA receptors to the synapse (Derkach et al., 1999, 2007). This may in turn impair LTP expression following EA, by limiting additional TBS-induced phosphorylation at Ser831. Thus, although the overall number of GluA1 subunits is decreased, the remaining GluA1 containing AMPA receptors are more likely found at synapses. Our observations early following EA contrast with the previously observed decrease in GluA1 Ser831 phosphorylation observed 3 h after pilocarpine induced seizures (Russo et al., 2013).

Phosphorylation of GluA1 at Ser845, a main target of PKA, was contrariwise decreased following both types of EA, and this decrease was more pronounced following ictal-like activity (Figure 6C). This change opposes the one observed after common TBS protocols *in vitro* (Park et al., 2021) but is consistent with our findings of decreased GluA1 containing receptors at synapses, an effect that may also reflect and endogenous neuroprotective measure against hyperexcitability induced by EA, as phosphorylation of GluA1 subunits at Ser845 drives AMPA receptor synaptic insertion, which could contribute to maladaptive homeostatic plasticity following seizures. This is fundamentally different from what is observed in the chronic period in the Li^2+^-pilocarpine model of epilepsy, where both GluA1 P831 and P845 phosphorylation levels are increased (Serpa et al., 2022).

This work also found a decrease in synaptic Kv4.2 channels following interictal-like EA but not following ictal-like EA (Figure 6F). Expression of Kv4.2 channels is more prominent in dendritic spines vs. dendritic shafts, being largely responsible for the Ca^2+^-activated delayed rectifying A-current (I_*A*_) in CA1 pyramidal neuron distal dendrites, where it acts to control signal propagation and compartmentalization (Kim et al., 2007; Beck and Yaari, 2008; Kim and Hoffman, 2008). LTP induction with strong TBS stimuli reduces Kv4.2 dendritic membrane levels, leading to a shift in the voltage-dependence of I_*A*_, and Kv4.2 channel activity contributes to LTP expression (Frick et al., 2004; Kim and Hoffman, 2008). Kv4.2 channels are responsible for the precision of the time window of pre and postsynaptic activity allowed for LTP induction (Zhao et al., 2011) and I_*A*_ activation also influences synaptic NMDA receptor composition at CA1 pyramidal neurons (Jung et al., 2008) and is synaptic morphology and seizure susceptibility (Tiwari et al., 2020). Altogether, our findings suggest that LTP will be easier to induce following interictal-like EA, resulting from an enlarged time window provided by Kv4.2 withdrawal from synapses. This may explain the lower impact and faster recovery of LTP expression following interictal-like vs ictal-like activity in our study, as this effect opposes the overall observed putative neuroprotective molecular remodeling at synapses (e.g., in AMPA receptor levels and phosphorylation). Being observed only following interictal-like (and not ictal-like EA), this effect is either not contributing to the putative protective effects of interictal-like EA (Avoli, 2001; Avoli et al., 2006) in epileptogenesis or reflects the ability of interictal-like activity to promote a faster synaptic recovery.

Synaptic α-tubulin levels were also altered in hippocampal slices following ictal-like and interictal-like EA (Figure 7F). As such, we could not use this protein as loading control for western blot analysis. Until recently the role of the microtubule cytoskeleton at synapses was not fully acknowledged but recent evidence has shed light on its importance in synaptic function, neurotransmitter release and synaptic plasticity (Waites et al., 2021). Decreased α-tubulin levels found in this work following EA likely indicate structural changes in synaptic morphology, as previously reported after seizures (Leite et al., 2005), and suggest that α-tubulin may undergo incorporation into microtubules following EA. Interestingly, the control of synaptic Ca^2+^ overload by microtubule dynamics has recently been reported (Vajente et al., 2019). Whether this mechanism may play a role endogenous neuroprotection against hyperexcitable states it remains to be established.

Chemical synapses are specialized structures composed of microdomains such as the presynaptic active zone and the postsynaptic density. Synaptic membranes, in turn, comprise nanodomains such as lipid rafts, liquid-ordered or gel-like membrane nanodomains segregated from the bulk membrane and enriched in cholesterol, sphingolipids and GPI-anchored proteins, deemed essential for dynamic synaptic signaling in processes like neurotransmitter release and neurotransmitter receptor membrane cycling (Allen et al., 2007; Borroni et al., 2016). The membrane proteins caveolin-1 and flotillin-1 characterize the two main types of lipid rafts found at synapses, caveolae and planar lipid rafts (Allen et al., 2007; Borroni et al., 2016), and play an active role in promoting the phase separation that sustains these lipid nanodomains (Allen et al., 2007; Frick et al., 2007), yet, lipid domain stability is also regulated by its lipid composition and lipid leaflet distribution (Sodero et al., 2011). In this work we observed an extremely marked decrease in the synaptic levels of caveolin-1 and flotillin-1 (Figures 7A, B), suggesting that EA is a strong disruptor of normal lipid raft stability and of trafficking mechanisms regulated by these proteins. Previous studies showed that enhanced excitatory neurotransmission induces a massive cholesterol loss, and this was implicated in endogenous neuroprotection against oxidative stress (Iannilli et al., 2011; Sodero et al., 2011). Furthermore, the interaction of postsynaptic anchoring proteins like PSD-95 and gephyrin and neurotransmitter receptors, like AMPA receptor, with lipid raft domains is regulated by synaptic activity through post-translational modifications like palmitoylation or GPI anchoring (Papadopoulos et al., 2015; Tulodziecka et al., 2016; Itoh et al., 2018). This may in turn disrupt the normal synaptic signaling through AMPA, NMDA and GABA_*A*_ receptors and impact synaptic plasticity mechanisms. Caveolin-1 was shown to enhance AMPA receptor binding capacity through direct inhibition of PLA2 activity (Gaudreault et al., 2004), thus suggesting that reduced caveolin levels may impair glutamatergic transmission and LTP expression. Caveolin-1 was also deemed essential in post-injury remodeling of the neuronal membrane following different insults (Gaudreault et al., 2005), such as brain injury by cerebral ischemia (Jasmin et al., 2007). Flotillin-1 in turn was implicated in glutamatergic synapse maturation, dendritic pruning and promotion of glutamate release. Flotillin is also involved in clathrin-independent endocytosis, by inducing membrane curvature and formation of plasma-membrane invaginations morphologically similar to caveolae (Frick et al., 2007) a process linked to neurotransmitter transporter internalization (Cremona et al., 2011). As such, decreased levels of flotillin-1 following seizures may contribute to decreased synaptic stability, impaired glutamatergic transmission and the impaired synaptic plasticity observed in this work. Altogether, this suggests that caveolins and flotillins are promising therapeutic targets to prevent epileptogenesis. The exact mechanism to be targeted requires still further investigation.

One possible explanation for the massive loss of some synaptic lipid raft markers could be the occurrence of massive synaptic protein internalization, degradation and autophagy in response to synaptic activity during hyperexcitable states (Li et al., 2012; Soykan et al., 2021). This would also imply a reduction in nerve terminal synaptic vesicles and its characteristic markers. Our observations that the synaptic vesicle marker synaptophysin is not significantly altered following both ictal-like and interictal-like EA (Figure 7C), suggests that these mechanisms are not likely contributing to reduce levels of synaptic proteins.

Distinct from classic lipid raft markers, the synaptic levels of postsynaptic scaffolding protein PSD-95, present at glutamatergic synapses, were moderately enhanced while the levels of gephyrin, present at GABAergic synapses, were massively enhanced following ictal-like and interictal-like EA (Figures 7D, E). PSD-95 is a lipid raft nucleating protein and plasma membrane cholesterol depleting was shown to reduce PSD-95 targeting to neuronal dendrites (Hering et al., 2003; Tulodziecka et al., 2016). Our results are in conflict with previous observations reporting that enhanced excitatory synaptic transmission causes cholesterol depleting (Sodero et al., 2011), expected to lower PSD-95 levels, and are also not in line with evidence in this work that synaptic lipid rafts are destabilized by EA and with reports that synaptic AMPA receptor levels, stabilized at synapses by palmitoylated PSD-95 (Han et al., 2015; Matt et al., 2019), are reduced following EA. However, this may reflect a specific response of the postsynaptic active zone to EA, since maintaining postsynaptic lipid rafts may be crucial to prevent epileptogenesis and abnormal spine growth by keeping together the molecular machinery allowing for AMPA receptor depalmitoylation and internalization (Han et al., 2015; Itoh et al., 2018).

GABAergic transmission and interneuron networks are key players in seizure initiation and in epileptogenesis (Magloire et al., 2019; Avoli et al., 2022). GABA_A_ receptor postsynaptic recruitment to the inhibitory GABAergic synapses requires the scaffold protein gephyrin, that establishes a dynamic lattice-like structure with nanodomains of different size and density (Pizzarelli et al., 2020), and the action of the guanine nucleotide exchange factor collybistin through a PI3P dependent-process (Papadopoulos et al., 2017). Collybistin mutants with reduced lipid binding affinity disrupt GABAergic synapses by preventing gephyrin synaptic clustering and GABA_A_ receptor targeting and this was in turn reported to trigger epilepsy (Papadopoulos et al., 2015). Gephyrin palmitoylation drives its membrane insertion and macromolecular clustering thus facilitating GABAergic transmission (Dejanovic et al., 2014; Matt et al., 2019). As such, the enhanced synaptic levels of gephyrin found in this work following EA may reflect its synaptic recruitment to reinforce GABAergic transmission, through GABA_A_ recruitment, as an endogenous neuroprotective effort against hyperexcitability during ictal-like and interictal-like EA (Barberis, 2020). A similar response of GABAergic synapses was observed following anoxia (Lushnikova et al., 2011). This contrasts with the decreased levels of PSD-95 and gephyrin found in animal models and human MTLE (Sun et al., 2009; Fang et al., 2011; Bento-Oliveira, 2018), reinforcing the idea that acute and chronic synaptic responses to seizures are distinct.

## Conclusion

Long term potentiation (LTP) expression is impaired following EA, and this impairment is stronger following ictal-like than after interictal-like activity. Altered LTP expression is accompanied by altered PPF, evidencing opposing presynaptic modifications of glutamate release, following ictal-like and interictal-like EA. In addition, we observed marked modifications in several synaptic proteins involved in synaptic plasticity mechanisms such as AMPA receptor subunits and its phosphorylation, and Kv4.2 channels. Furthermore, we detected altered synaptic membrane structure and domain regulating proteins like caveolin-1, flotillin-1, PSD-95 and gephyrin. Altogether, these results suggest that early changes in synaptic plasticity are a promising target for antiepileptogenic therapies and strategies focusing on modulation of synaptic lipid raft domain dynamics may prove relevant to prevent epileptogenesis.

## Data availability statement

The raw data supporting the conclusions of this article will be made available by the authors, without undue reservation.

## Ethics statement

This animal study was reviewed and approved by the Ethics Committee of the Faculty of Medicine, University of Lisbon (Comissão de Ética para a Saúde do CHLN/FMUL). Approval was given in writing based on a detailed procedure report by the authors.

## Author contributions

JC-R, NR, and AS-C: formal analysis, methodology. SV: methodology. DC-R: formal analysis, methodology, resources, supervision, funding acquisition, project administration, writing—original draft, review, and editing. All authors contributed to the article and approved the submitted version.
